# Function of IRAG2 Is Modulated by NO/cGMP in Murine Platelets

**DOI:** 10.3390/ijms23126695

**Published:** 2022-06-15

**Authors:** Sally Prüschenk, Jens Schlossmann

**Affiliations:** Department of Pharmacology and Toxicology, Institute of Pharmacy, University of Regensburg, 93040 Regensburg, Germany; sally.prueschenk@chemie.uni-regensburg.de

**Keywords:** cGKI, cGMP, IP_3_R, IRAG, IRAG1, IRAG2, Jaw1, LRMP, phosphorylation, PKGI, platelet aggregation, platelets

## Abstract

Inositol 1,4,5-triphosphate receptor-associated 2 (IRAG2) is a type II membrane protein located at the endoplasmic reticulum. It is a homologue of inositol 1,4,5-triphosphate receptor-associated cGMP kinase substrate 1 (IRAG1), a substrate protein of cGMP-dependent protein kinase I (PKGI), and is among others expressed in platelets. Here, we studied if IRAG2 is also located in platelets and might be a substrate protein of PKGI. IRAG2 was detected in platelets of IRAG2-WT animals but not in those of IRAG2-KO animals. Next, we validated by co-immunoprecipitation studies that IRAG2 is associated with IP_3_R1-3. No direct stable interaction with PKGIβ or with IRAG1 was observed. Phosphorylation of IRAG2 in murine platelets using a Ser/Thr-specific phospho-antibody was found in vitro and ex vivo upon cGMP stimulation. To gain insight into the function of IRAG2, platelet aggregation studies were performed using thrombin and collagen as agonists for treatment of isolated IRAG2-WT or IRAG2-KO platelets. Interestingly, platelet aggregation was reduced in the absence of IRAG2. Pretreatment of wild type or IRAG2-KO platelets with sodium nitroprusside (SNP) or 8-pCPT-cGMP revealed a further reduction in platelet aggregation in the absence of IRAG2. These results show that IRAG2 is a substrate of PKGI in murine platelets. Furthermore, our results indicate that IRAG2 is involved in the induction of thrombin- or collagen-induced platelet aggregation and that this effect is enhanced by cGMP-dependent phosphorylation of IRAG2. As IRAG1 was previously shown to inhibit platelet aggregation in a cGMP-dependent manner, it can be speculated that IRAG2 exerts an opposing function and might be an IRAG1 counterpart in murine platelets.

## 1. Introduction

Platelet activation and aggregation upon vascular injury is essential for primary hemostasis, but also has a function in atherogenesis and formation of occlusive thrombi [1]. The gaseous molecule nitric oxide (NO) is an endogenous inhibitor of platelet aggregation by activating soluble guanylyl cyclase (sGC) and therefore leading to an increase in cyclic guanosine monophosphate (cGMP) production [2,3,4,5]. cGMP induces a PKGIβ-dependent phosphorylation of the inositol 1,4,5-triphosphate receptor-associated cGMP kinase substrate 1 (IRAG1), resulting in an inhibition of inositol 1,4,5-trisphosphate (IP_3_)-induced Ca^2+^ release from the endoplasmic reticulum (ER) [6,7]. It is shown that the formation of a macro complex consisting of IRAG1, IP_3_R1 and PKGIβ mediates the NO/cGMP-dependent inhibition of thrombin-induced Ca^2+^ release in platelets and is also an important factor of NO/cGMP-dependent inhibition of platelet aggregation [8,9].

Inositol 1,4,5-triphosphate receptor-associated 2 (IRAG2) is a type II membrane protein localized to the cytoplasmic face of the ER and was first described in 1994 by Behrens et al. [10,11]. IRAG2 is also known as Jaw1 or lymphoid-restricted membrane protein (LRMP) and shows a homology with IRAG1, especially in its coiled coil domain with a homology of 44% [12,13,14]. The coiled coil domain of IRAG1 is essential for interaction with inositol trisphosphate receptors (IP_3_ receptors/IP_3_R) and regulation of Ca^2+^ release from intracellular stores [6,15]. IRAG2 was shown to interact with IP_3_R3 via its coiled coil domain in COS7 heterologous expression system and also with IP_3_R2 in intestinal tuft cells [13,16]. Recently, we observed an interaction of IRAG2 with IP_3_R1-3 in the pancreas [17]. Expression of IRAG2 was found in a variety of tissues and cell lines, e.g., B-cell lines, T-cell lines, spleen, thymus, sinoatrial nodes, sweet, bitter and umami taste-responsive cells or intestinal tuft cells [10,12,13,16,18]. Recently, we also detected IRAG2 expression in pancreatic acinar cells [17].

The physiological and molecular functions of IRAG2 are still widely unknown. Until now, in platelets no data exist regarding the expression and functional role of IRAG2 on platelet aggregation. Due to its homology to IRAG1 [12,13,14], it is possible that IRAG2 has similar interaction partners in platelets and that IRAG2 also has an effect on agonist induction or NO/cGMP-dependent inhibition of platelet aggregation. Furthermore, it is unknown if IRAG2 is phosphorylated by cGMP via PKGI in platelets similar to its homologue IRAG1 and if it thereby modulates NO/cGMP-dependent platelet inhibition.

In our study, we analyzed the expression of IRAG2 in platelets and its interaction with IP_3_ receptors as well as its possible role in agonist-induced and NO/cGMP-dependent inhibition of platelet aggregation.

## 2. Results

### 2.1. Expression of IRAG2 in Murine Platelets

**Figure 1:**
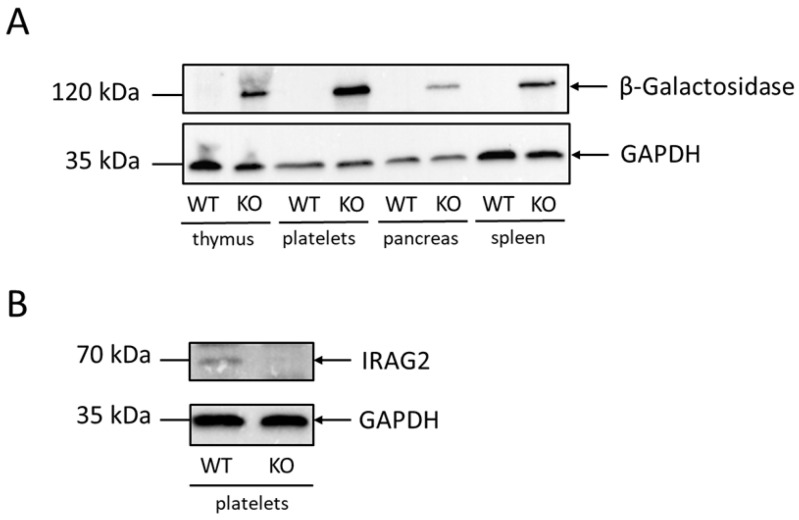
Expression of IRAG2 in murine platelets. (**A**) Western Blot analysis revealed expression of β-Galactosidase as a reporter for IRAG2 in thymus, platelets, pancreas and spleen from lacZ × IRAG2-KO mice (KO); no signal was detected in tissues from IRAG2-WT mice (WT). (**B**) Expression of IRAG2 was confirmed by Western Blotting using an IRAG2-specific antibody. IRAG2 was detected in platelets from IRAG2-WT mice (WT) but not in platelets from IRAG2-KO mice (KO). GAPDH was used as a loading control (**A**,**B**).

The expression pattern of IRAG2 was analyzed via Western Blotting in different tissues using lacZ × IRAG2-KO mice, which express β-Galactosidase in IRAG2-KO animals under control of the IRAG2 promotor and therefore act as a reporter for IRAG2. We detected expression of β-Galactosidase in platelets from lacZ × IRAG2-KO mice (Figure 1A) but not in platelets from IRAG2-WT mice (Figure 1A), indicating that IRAG2 is expressed in murine platelets. As a positive control, spleen, thymus and pancreas were analyzed for expression of β-Galactosidase (Figure 1A), since IRAG2 has been previously detected in these tissues [10,17]. Expression of IRAG2 in platelets was confirmed with an IRAG2-specific antibody in platelets from IRAG2-WT mice. However, no signal was detected in platelets from IRAG2-KO mice (Figure 1B). Taken together, these data revealed IRAG2 expression in murine platelets.

### 2.2. Interaction of IRAG2 with IP_3_R1-3

**Figure 2:**
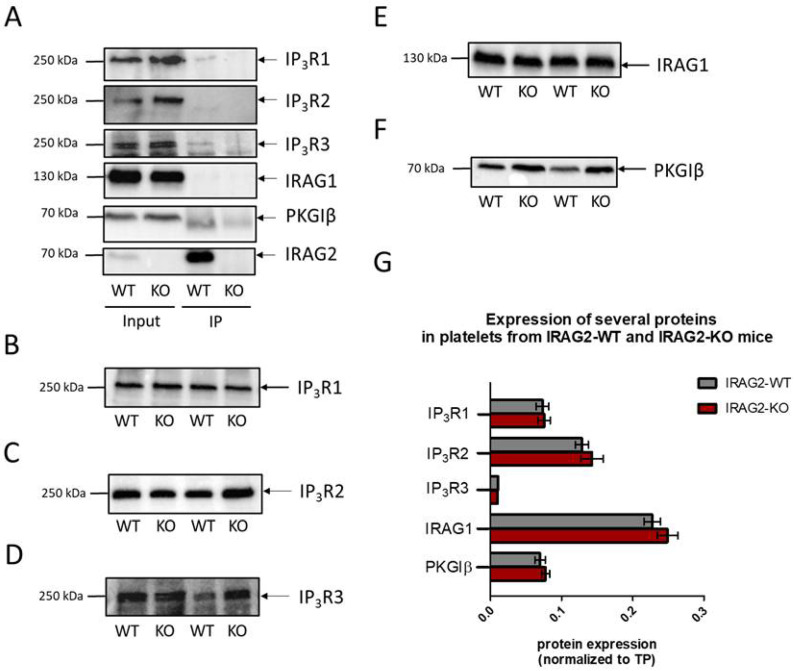
Interaction of IRAG2 with IP_3_ receptors (**A**) and expression of several proteins in platelets from IRAG2-WT (WT) and IRAG2-KO (KO) mice (**B**–**G**). (**A**) Immunoprecipitation of IRAG2 with IRAG2-specific antibody and Western Blot analysis of co-immunoprecipitated interaction partners. An amount of 70 µg of protein was used as input probe and 1000 µg of protein for immunoprecipitation. (**B**–**F**) Representative Western Blots for protein expression of IP_3_R1-3, IRAG1 and PKGIβ. (**G**) Signal intensity of each band was quantified and normalized to total protein (TP) of the appropriate Lane for quantification. Expression of IP_3_R1 (WT: *n* = 11; KO: *n* = 11), IP_3_R2 (WT: *n* = 5; KO: *n* = 5), IP_3_R3 (WT: *n* = 5; KO: *n* = 5), IRAG1 (WT: *n* = 11; KO: *n* = 11) and PKGIβ (WT: *n* = 17; KO: *n* = 17) was not altered between platelets from IRAG2-WT (WT) and IRAG2-KO (KO) mice and statistical analysis of protein expression revealed no significant differences between IRAG2-WT and IRAG2-KO platelets. Images of TP are shown in Supplementary Materials Figure S1, and graphs are shown as mean ± SEM.

The interaction of IRAG2 with different IP_3_ receptor subtypes has been shown in cell lines as well as in murine tissues. In COS7 heterologous expression system interaction of IRAG2 with IP_3_R3 was shown [13]. Recently, we detected the interaction of IRAG2 with IP_3_ receptor types 1, 2 and 3 in the murine pancreas [17]. IRAG1 has been shown to form a macro complex with IP_3_R1 and PKGIβ in murine platelets [8]. To test whether IRAG2 also interacts with IP_3_ receptors or PKGIβ in murine platelets, IRAG2 was immunoprecipitated with the IRAG2 antibody and co-immunoprecipitated interaction partners were analyzed by Western Blot. Interaction was detected with IP_3_R1, IP_3_R2 and IP_3_R3 in IRAG2-WT and no signal was detected in IRAG2-KO platelets (Figure 2A). However, the molecular weight of IP_3_R2 in immunoprecipitated probes of IRAG2-WT platelets appeared to be lower than the molecular weight for IP_3_R2 in the input probes of IRAG2-WT and IRAG2-KO. We detected no interaction of IRAG2 with PKGIβ in murine platelets. The Western Blot images of PKGIβ revealed an unspecific band in co-immunoprecipitated probes of IRAG2-WT and also of IRAG2-KO platelets, which appeared to be lower than the input bands for PKGIβ. This unspecific band is not a PKGIβ signal but is derived from the IRAG2 antibody that was used for co-immunoprecipitation (data not shown). In addition, no direct interaction of IRAG1 and IRAG2 was seen. To confirm that IRAG2 does not interact with IRAG1 directly, co-immunoprecipitation was also conducted with IRAG1 antibody in platelets from IRAG2-WT and IRAG2-KO mice. No signal for IRAG2 neither in IRAG2-WT nor in IRAG2-KO was observed when immunoprecipitating IRAG1 with the IRAG1-specific antibody (Supplementary Materials, Figure S2).

To test whether a knockdown of IRAG2 leads to different protein expression of the IP_3_ receptor subtypes, IRAG1 or PKGIβ, we analyzed expression of these proteins in lysates of IRAG2-WT and IRAG2-KO platelets by Western Blot. Expression of IP_3_R1 (Figure 2B,G), IP_3_R2 (Figure 2C,G), IP_3_R3 (Figure 2D,G), IRAG1 (Figure 2E,G) and PKGIβ (Figure 2F,G) was not altered in platelets from IRAG2-KO mice compared to IRAG2-WT mice.

### 2.3. Phosphorylation of IRAG2 by PKGI

**Figure 3:**
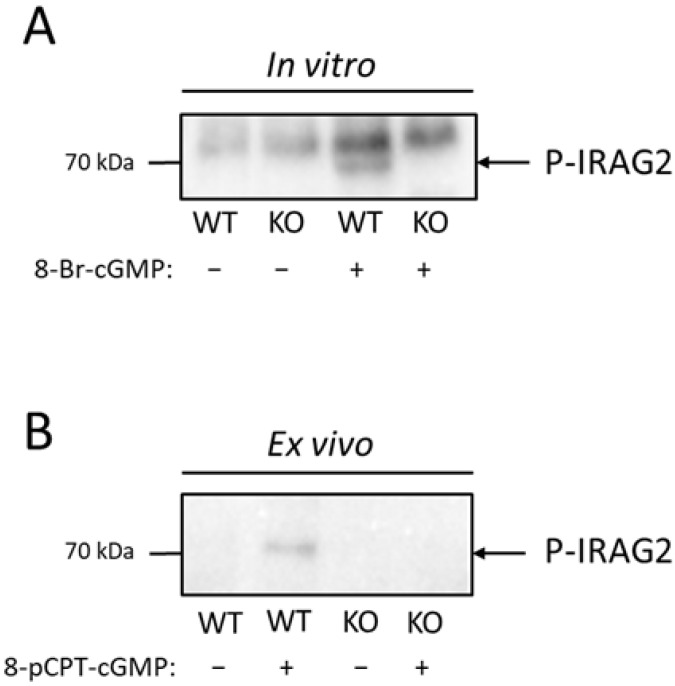
Phosphorylation of IRAG2 in platelets in vitro and ex vivo. (**A**,**B**) Platelets of IRAG2-WT (WT) and IRAG2-KO (KO) mice were stimulated in vitro with 100 µM 8-Br-cGMP (**A**) or ex vivo in intact platelets with 100 µM 8-pCPT-cGMP (**B**) for 20 min. Immunoprecipitation was performed to detect IRAG2-specific phosphorylation. Detection of IRAG2 phosphorylation was carried out by a phospho-(Ser/Thr) PKA substrate antibody. IRAG2 phosphorylation (P-IRAG2) was observed in vitro in lysed platelets (**A**) as well as ex vivo in intact platelets (**B**) from IRAG2-WT (WT) mice that were stimulated with cGMP. No signal was seen neither in unstimulated platelets of IRAG2-WT (WT) mice nor in stimulated or unstimulated platelets of IRAG2-KO (KO) mice (**A**,**B**).

It has been shown before that IRAG1 is phosphorylated by PKGIβ in murine platelets [8]. Phosphorylation of IRAG1 in platelets by activating the NO/cGMP pathway leads to an inhibition of Ca^2+^ release from the ER and therefore to an inhibition of platelet aggregation [8,9]. IRAG2 showed no direct interaction with PKGIβ (Section 2.2); however, it is possible that IRAG2 is phosphorylated in a cGMP-dependent manner by activation of PKGI. To test whether IRAG2 is phosphorylated via PKGI in platelets, we stimulated activation of PKGI and therefore phosphorylation of IRAG2 with 100 µM 8-Br-cGMP or 100 µM 8-pCPT-cGMP for 20 min in vitro in lysed platelets as well as ex vivo in intact platelets. Following incubation with cGMP or H_2_O as an unstimulated control, immunoprecipitation with the IRAG2 antibody was carried out to investigate IRAG2-specific phosphorylation. IRAG2 phosphorylation was detected using a phospho-(Ser/Thr) PKA substrate antibody that detected PKG-, PKA- or PKC-dependent phosphorylation at serine or threonine residues. When stimulating lysates of IRAG2-WT and IRAG2-KO platelets with 8-Br-cGMP, we detected phosphorylation of immunoprecipitated IRAG2. However, no phosphorylation was found in unstimulated and stimulated lysates of IRAG2-KO or unstimulated control probes of IRAG2-WT (Figure 3A). The same effect was seen in intact platelets upon stimulation with 100 µM 8-pCPT-cGMP. Phosphorylation was revealed in stimulated platelets of IRAG2-WT, but no signal was obtained neither in unstimulated platelets of IRAG2-WT mice nor in stimulated or unstimulated platelets of IRAG2-KO mice (Figure 3B). Taken together, we observed cGMP-dependent phosphorylation of IRAG2 in lysed as well as in intact platelets of IRAG2-WT mice.

### 2.4. Role of IRAG2 in Agonist-Induced Platelet Aggregation

**Figure 4:**
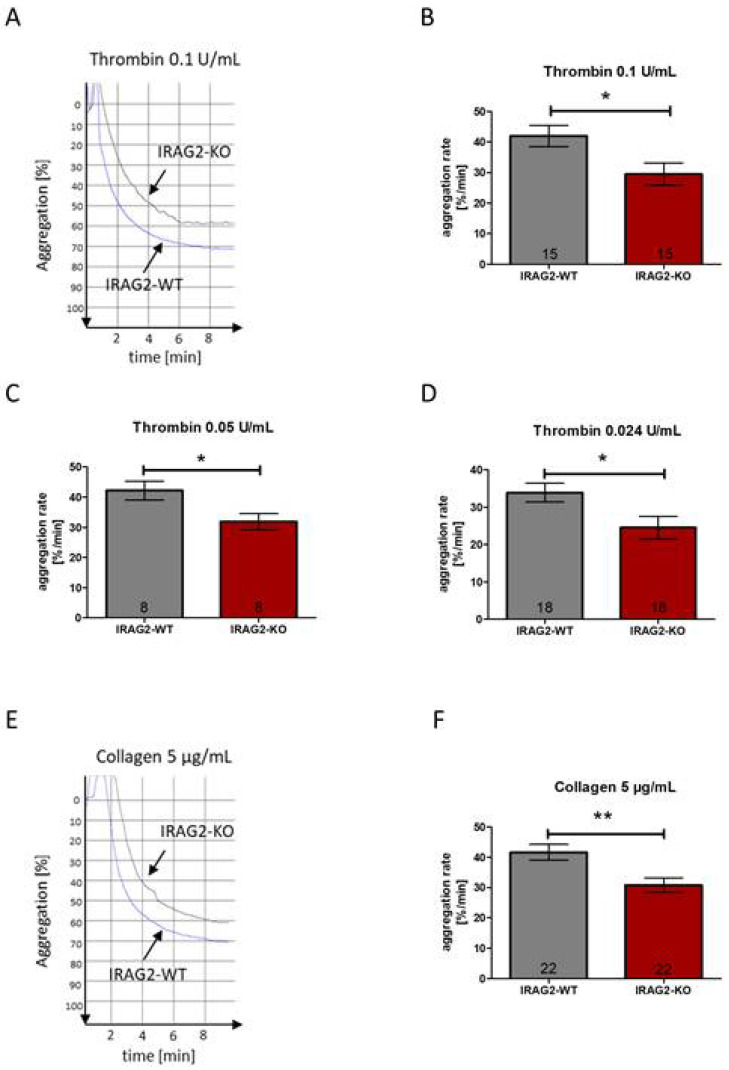
Agonist-induced aggregation of platelets from IRAG2-WT and IRAG2-KO mice. (**A**) Representative traces for aggregation of IRAG2-WT and IRAG2-KO platelets are shown for stimulation with thrombin 0.1 U/mL, where IRAG2-KO platelets revealed a lower aggregability compared to IRAG2-WT platelets. (**B**–**D**) Statistical evaluation of aggregability revealed a significantly lower aggregation rate for platelets from IRAG2-KO mice compared to platelets from IRAG2-WT mice upon stimulation with thrombin 0.1 U/mL (**B**), thrombin 0.05 U/mL (**C**) and thrombin 0.024 U/mL (**D**). (**E**,**F**) Representative traces for aggregation of IRAG2-WT and IRAG2-KO platelets upon stimulation with collagen 5 µg/mL (**E**) and statistical evaluation of aggregation rate upon stimulation with collagen 5 µg/mL (**F**) showed a significantly lower aggregability of IRAG2-KO platelets compared to IRAG2-WT platelets. Aggregation rate is calculated as maximal slope of the curve. For each aggregation 1.0 × 10^8^ platelets/mL were used. Number of performed experiments (number of mice) are shown in the graphs and mean ± SEM is shown by bars. Significant differences are indicated as (*) (*p* < 0.05) and (**) (*p* < 0.01).

It has been shown before that IRAG1 affects platelet aggregation [8,9]. The aggregation rate of platelets from IRAG1-KO mice reveals a significantly higher aggregability compared to platelets from IRAG1-WT mice [9]. The homology of IRAG1 and IRAG2 [12,13,14] raises the possibility that IRAG2 also affects agonist-induced platelet aggregation. To test whether IRAG2 has an effect on agonist-induced platelet aggregation, platelets of IRAG2-WT and IRAG2-KO mice were stimulated with different agents that induce platelet aggregation. Aggregability of IRAG2-WT and IRAG2-KO platelets was measured by determining the maximal slope of the aggregation curves, which reflects the maximal aggregation rate of the platelets upon stimulation. Platelet aggregation was evoked by using thrombin 0.1 U/mL, thrombin 0.05 U/mL and thrombin 0.024 U/mL or collagen 5 µg/mL. All experiments were conducted with the same number of platelets (each 1.0 × 10^8^/mL) for both genotypes. Despite using the same amount of IRAG2-WT and IRAG2-KO platelets, for all used thrombin concentrations, platelet aggregation was significantly lower in IRAG2-KO platelets compared to IRAG2-WT platelets (Figure 4A–D). The same effect was detected on collagen-induced platelet aggregation. When stimulating the platelets with collagen 5 µg/mL, platelet aggregation was significantly lower in platelets from IRAG2-KO mice compared to platelets from IRAG2-WT mice (Figure 4E,F). Taken together, these data revealed a lower agonist-induced aggregability of platelets from IRAG2-KO mice compared to platelets from IRAG2-WT mice.

### 2.5. Role of IRAG2 in NO/cGMP-Dependent Inhibition of Platelet Aggregation

**Figure 5:**
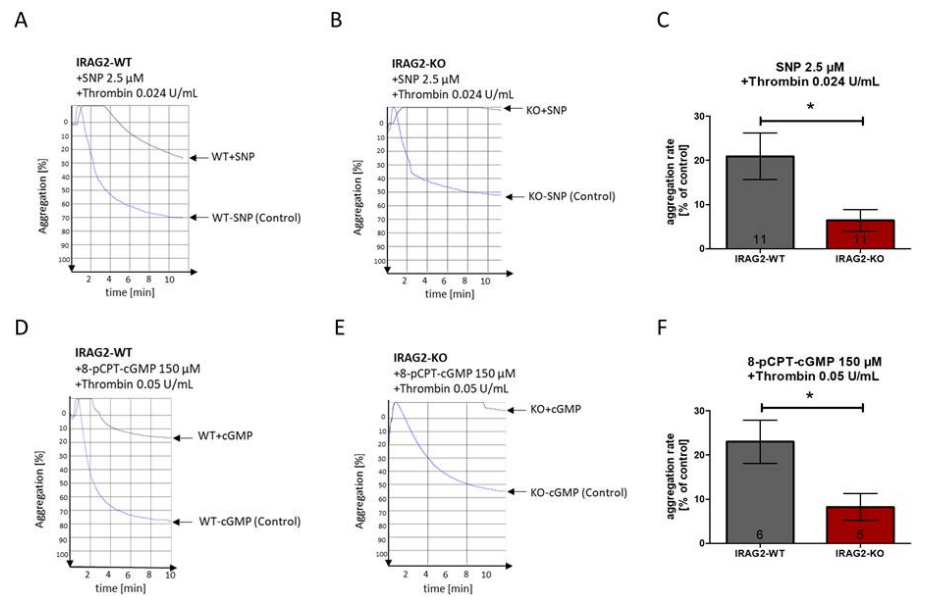
NO/cGMP-dependent inhibition of platelet aggregation in platelets from IRAG2-WT and IRAG2-KO mice. Aggregation rate was calculated as maximal slope and aggregation rate with inhibitor was calculated as % of control (control: aggregation rate without inhibitor). (**A**,**B**) Representative images of inhibition of platelet aggregation in platelets from IRAG2-WT mice (**A**) and IRAG2-KO mice (**B**) using sodium nitroprusside (SNP) 2.5 µM as an inhibitor and thrombin 0.024 U/mL to evoke platelet aggregation. (**C**) Statistical evaluation of platelet inhibition with SNP 2.5 µM revealed reduced aggregation rate as % of control in platelets from IRAG2-KO mice compared to IRAG2-WT mice. (**D**,**E**) Representative images of platelet inhibition in platelets from IRAG2-WT mice (**D**) and IRAG2-KO mice (**E**) with 8-pCPT-cGMP 150 µM as an inhibitor of platelet aggregation and thrombin 0.05 U/mL as an agonist of aggregation. (**F**) Statistical evaluation showed a lower aggregation rate as % of control in platelets from IRAG2-KO mice compared to platelets from IRAG2-WT mice. For each aggregation 1.0 × 10^8^ platelets/mL were used. Number of experiments (number of mice) are shown in the graphs and mean ± SEM are indicated by bars. Significant differences are shown by (*) (*p* < 0.05).

IRAG1 is known to form a ternary complex with IP_3_R1 and PKGIβ [8]. Activation of the NO/cGMP pathway causes a phosphorylation of IRAG1 via PKGIβ and leads to an inhibition of Ca^2+^ release from the endoplasmic reticulum and therefore to an inhibition of platelet aggregation [6,8,9]. The aggregation rate of mice lacking IRAG1 is hardly affected upon stimulation with 8-pCPT-cGMP or only weakly inhibited by NO donors [8,9]. IRAG2 also interacts with IP_3_ receptors in platelets (Section 2.2) and is phosphorylated upon stimulation with cGMP-analogues (Section 2.3), though there is no direct interaction with PKGIβ (Section 2.2). To investigate whether IRAG2 has an effect on NO/cGMP-dependent inhibition of platelet aggregation, platelets (each 1.0 × 10^8^/mL) were preincubated with 8-pCPT-cGMP (150 µM, 20 min) or the NO donor natrium nitroprusside (SNP; 2.5 µM, 2 min). Following preincubation with the inhibitor, platelet aggregation was evoked by using thrombin (0.05 U/mL or 0.024 U/mL) or collagen (5 µg/mL). Aggregation rate was calculated as maximal slope, and aggregability upon incubation with the inhibitor was calculated as aggregation rate in percent (%) of control (control: agonist-induced aggregation rate without inhibitor). When inhibiting platelet aggregation with SNP 2.5 µM, the aggregation rate (% of control) was significantly lower in platelets from IRAG2-KO mice compared to IRAG2-WT mice (Figure 5A–C). The same effect was seen when preincubating platelets with 8-pCPT-cGMP (150 µM), where platelets from IRAG2-KO revealed a significantly lower aggregation rate (% of control) compared to platelets from IRAG2-WT mice (Figure 5D–F). Only a weak tendency for a reduced aggregation rate (% of control) of IRAG2-KO platelets compared to IRAG2-WT platelets was seen for the inhibition of platelet aggregation with 8-pCPT-cGMP (200 µM) or SNP (2.5 µM) when using collagen (5 µg/mL) as an agonist (Supplementary Materials, Figure S3); however, this effect was not significant. Taken together, the aggregation rate (% of control) was decreased in IRAG2-KO platelets compared to IRAG2-WT platelets when using SNP (2.5 µM) or 8-pCPT-cGMP (150 µM) as an inhibitor of platelet aggregation. Therefore, inhibition of platelet aggregation by SNP or 8-pCPT-cGMP was enhanced in platelets from IRAG2-KO mice compared to platelets from IRAG2-WT mice.

## 3. Discussion

Inositol 1,4,5-triphosphate receptor-associated 2 (IRAG2) is also known as Jaw1 or lymphoid-restricted membrane protein (LRMP). It contains a coiled coil domain and a COOH-terminal hydrophobic anchor domain, which is important for the localization to the endoplasmic reticulum (ER) [10,11]. Recently, we detected expression of IRAG2 in pancreatic acinar cells, where it interacts with all types of IP_3_ receptors and modulates basal calcium levels and basal secretion of amylase [17]. IRAG2 has been detected before in a variety of other tissues and cell lines, since its first description in 1994 by Behrens et al. [10]. Expression was reported, e.g., in thymus, spleen, B- and T-cell lines, sinoatrial nodes or sweet, bitter and umami taste-responsive cells [10,12,13,18]. IRAG2 shares a homology with inositol 1,4,5-triphosphate receptor-associated cGMP kinase substrate 1 (IRAG1) [12,13,14], that is expressed among others in human and in murine platelets [8]. In our current study, we demonstrate that IRAG2 is, like its homologue, also expressed in murine platelets. Until now, the function of IRAG2 in murine platelets was unknown. However, this expression suggests that IRAG2 might be involved in platelet function, e.g., platelet aggregation. In addition, to our knowledge, this is the first time that the expression of IRAG2 is shown in murine platelets.

It is reported that IRAG2 shares a homology with IRAG1, especially in its coiled coil domain with 44% [12,13,14]. IRAG1 forms a macro complex with the IP_3_R1 and PKGIβ in platelets, where the coiled coil domain is essential for the interaction with the IP_3_R1 [6,8]. Recently, we detected an interaction of IRAG2 with IP_3_R1-3 in the murine pancreas [17]. In addition, an interaction of IRAG2 with different IP_3_ receptor subtypes has been detected before in other tissues. In intestinal tuft cells, IRAG2 interacts with IP_3_R2 [16], and in COS7 heterologous expression system interaction has been detected with IP_3_R3 [13]. Therefore, it is possible that this interaction with IP_3_ receptors also occurs in platelets. Here, we show that IRAG2 interacts with IP_3_R1, IP_3_R2 and IP_3_R3 in murine platelets. However, in the input platelet lysates IP_3_R2 shows a higher molecular weight compared to the co-immunoprecipitated IP_3_R2 in IRAG2-WT probes, but we observe no signal in the co-immunoprecipitated probes of IRAG2-KO. Therefore, we assume that this is not an unspecific band for IP_3_R2, but that IRAG2 interacts with a shorter fragment of the IP_3_R2, which is only detectable after immunoprecipitating the complex of IRAG2 and IP_3_R2. In addition, the amount of co-immunoprecipitated IP_3_R1-3 is low, and therefore the interaction of IRAG2 and IP_3_R1-3 appears to be weak. Furthermore, we investigated if IRAG2 directly interacts with IRAG1 or PKGIβ. In our study, we did not see a direct interaction of IRAG2 neither with PKGIβ nor with IRAG1. Hence, IRAG2 does not form a macro complex in platelets, where PKGIβ or IRAG1 are included. This is not surprising, as the stable interaction site of IRAG1 with the leucine zipper of PKGIβ is located between amino acid 152 and 184 of the N-terminal part of IRAG1, which is lacking in IRAG2 [7,19].

Our finding that IRAG2 reveals no direct interaction with PKGIβ does not exclude a cGMP-dependent phosphorylation of IRAG2 via PKGI. We stimulated lysed platelets as well as intact platelets with the cGMP analogue 8-pCPT-cGMP and thereby detected phosphorylation of IRAG2 in murine platelets. To evaluate this phosphorylation, we used a phospho-(Ser/Thr) PKA substrate antibody that identifies PKA-, PKC- or PKG-dependent phosphorylation at serine or threonine residues of proteins. As cGMP or cGMP analogues such as 8-pCPT-cGMP predominantly activate PKGI [4,20], IRAG2 phosphorylation after stimulation with 8-pCPT-cGMP might be mediated mainly through PKGI. To detect which isoform of PKGI, PKGIα or PKGIβ, is responsible for the phosphorylation of IRAG2, further experiments are required. However, in murine platelets it is known that PKGIβ is the predominant isoform and only a small amount of PKGIα is expressed. In human platelets, only PKGIβ is expressed. In addition, IRAG1 phosphorylation is mediated through PKGIβ in murine and in human platelets [8]. This raises the possibility that IRAG2 phosphorylation in murine platelets is also achieved through PKGIβ. Our result, that IRAG2 is phosphorylated in a cGMP-dependent manner in murine platelets, correlates with the findings of Makhoul et al., who analyzed phosphoproteins in human platelets by a quantitative phosphoproteomics study. They detected phosphorylation of LRMP at amino acids Ser363, Thr375 and Ser418 upon stimulation with NO donors or riociguat in human platelets [21]. Remarkably, the phosphorylation sites of Ser363 and Thr375 in IRAG2 (consensus sequences: RSAS^363^ and RRVT^375^) are very homologous to the identified phosphorylation sites of Ser664 and Ser677 in IRAG1 (consensus sequences: RSMS^664^ and RRVS^677^). For future studies, it is also necessary to detect the exact phosphorylation sites of IRAG2 in murine platelets.

In IRAG1-KO mice, platelet aggregation upon stimulation with agonists such as thrombin, collagen or the thromboxane A2 (TxA2) analogue U46619 is increased compared to IRAG1-WT mice [9]. These agonists stimulate platelet aggregation mainly by activating the phospholipase C (PLC) and therefore generating inositol trisphosphate (IP_3_), which leads to an increase in cytosolic Ca^2+^ [22,23]. This increase in Ca^2+^ results in an activation of GPIIb/IIIa and thus to the aggregation of platelets mediated by fibrinogen [23,24]. In our study, we used collagen and thrombin to evoke platelet aggregation to examine the effect of IRAG2 deficiency in platelets. In contrast to IRAG1-KO platelets, IRAG2-KO platelets revealed a lower aggregation rate compared to WT mice. Consequently, IRAG2 augments agonist-induced platelet aggregation and therefore exerts an opposing function on IRAG1. Opposing functions of IRAG1 and IRAG2 have also been observed before by Peters et al., where IRAG1 and IRAG2 both interact with HCN4 channels, but revealed contrary effects on the HCN4 function [12]. To analyze if the cGMP-dependent phosphorylation of IRAG2 impacts platelet aggregation, we preincubated the platelets with the NO donor natrium nitroprusside (SNP) or the cGMP analogue 8-pCPT-cGMP. NO donors or cGMP analogues cause an inhibition of platelet aggregation [2,3,4,20]. This effect is mediated by phosphorylation of IRAG1 via PKGI, leading to a reduced secretion of Ca^2+^ from the ER and therefore to an inhibition of platelet aggregation [6,8,9]. For IRAG2, we also observed opposing effects compared to IRAG2 on the inhibition of platelet aggregation. IRAG1-KO mice show no inhibition or only a weak inhibition of platelet aggregation after preincubation with SNP or 8-pCPT-cGMP [9]. In contrast, for IRAG2 we observe a reduced aggregation rate and therefore an enhanced inhibition of platelet aggregation in platelets from IRAG2-KO mice. This leads to the assumption that phosphorylation of IRAG2 by activation of the NO/cGMP pathway enhances platelet aggregation and that IRAG2 might be the counterpart to IRAG1 in murine platelets. Future experiments will require the measurement of Ca^2+^ in IRAG2-KO platelets to see if the reduced platelet aggregation in IRAG2-KO platelets occurs due to lower Ca^2+^ release after phosphorylation of IRAG2 by activation of the NO/cGMP pathway. In addition, it will be interesting to investigate if IRAG2 might have an impact on thrombus formation after vascular injury. For IRAG1 it is seen that it prevents thrombus formation in a NO/cGMP-dependent manner [8]. As IRAG2 appears to enhance aggregability of platelets, IRAG2 might have a potential role in the formation of thrombi and therefore in atherogenesis and development of cardiovascular diseases.

In summary, our study reveals new outcomes about the function of IRAG2 in platelets:

1. IRAG2 is expressed in murine platelets and interacts with IP_3_R1, IP_3_R2 and IP_3_R3; however, no stable interaction of IRAG2 is observed with PKGIβ or IRAG1. Hence, no macro complex consisting of IRAG2, PKGIβ and IP_3_R1 is formed like it is reported for IRAG1.
2. IRAG2 is phosphorylated in a cGMP-dependent manner by PKGI.
3. IRAG2-KO mice reveal a reduced platelet aggregation rate compared to IRAG2-WT mice, indicating that IRAG2 enhances platelet aggregation. When preincubating platelets with SNP or 8-pCPT-cGMP, the aggregation rate upon treatment with thrombin is reduced in platelets of IRAG2-KO mice compared to IRAG2-WT mice. Therefore, the inhibition of platelet aggregation by SNP or cGMP is enhanced in IRAG2-KO.

Taken together, IRAG2 appears to act as a counterpart to IRAG1 in murine platelets with opposing effects on platelet aggregation.

## 4. Materials and Methods

### 4.1. Animals

Global *Irag2*^−/−^ and *lacZ* × *Irag2*^−/−^ mice were generated as previously described [17]. All experiments were conducted with male and female IRAG2-KO mice (genotype: *Irag2*^−/−^), lacZ × IRAG2-KO mice (genotype: *lacZ* × *Irag2*^−/−^) and their wild type littermates (IRAG2-WT; genotype: *Irag2^+/+^*, *Irag2^fl/fl^*) older than 8 weeks. Animals were bred and maintained in the animal facilities of the University of Regensburg according to the Guidelines of the Federation of European Laboratory Animal Science Associations (Bavaria, Germany; Regierung von Unterfranken: DMS 2532-2-236), following all guidelines according to the German animal protection law, with free access to food and water ad libitum.

### 4.2. Materials

The following chemicals and antibodies were purchased for this study: 8 pCPT-cGMP, 8-Br-cGMP (BIOLOG Life Science Institute, Bremen, Germany), sodium nitroprusside (SNP; Sigma Aldrich^®^, Taufkirchen, Germany), collagen, thrombin (Probe & Go, Osburg, Germany), anti-mouse LRMP (C-terminal) = IRAG2-antibody (Sigma-Aldrich^®^, Taufkirchen, Germany; SAB1306900), IP_3_R1-antibody (Novusbio, Abingdon, UK; NBP1-21398), IP_3_R2-antibody (Santa Cruz, Heidelberg, Germany; sc-7278), IP_3_R3-antibody (BD Transduction Laboratories TM, BD Biosciences, San Jose, CA, USA; 610313), β-Galactosidase-antibody (Abcam plc, Cambridge, UK; ab9361), Phospho-(Ser/Thr) PKA substrate-antibody (Cell Signaling Technology, Leiden, The Netherlands; #9621), GAPDH-antibody (Cell Signaling Technology, Leiden, The Netherlands; #2118), secondary antibody goat-anti-mouse (Sigma-Aldrich^®^, Taufkirchen, Germany; A4416), secondary antibody goat-anti-rabbit (Dianova GmbH, Hamburg, Germany; 111-035-003), secondary antibody donkey-anti-goat (Dianova GmbH, Hamburg, Germany; 705-035-003), secondary antibody goat-anti-chicken (Dianova GmbH, Hamburg, Germany; DAB87304). The following antibodies are in-house productions: IRAG1-antibody, PKGIβ-antibody [25].

### 4.3. Isolation and Preparation of Platelets

Platelets of IRAG2-WT and IRAG2-KO or lacZ × IRAG2-KO mice were isolated as described before [9]. Briefly, mice were euthanized with CO_2_ and blood was drawn by cardiac puncture into 200 µL Alsever’s solution (Sigma Aldrich^®^, Taufkirchen, Germany), followed by mixing with 500 µL of buffer B (20 mM HEPES, 138 mM NaCl, 2.9 mM KCl, 1 mM MgCl_2_, 0.36 mM NaH_2_PO_4_, pH 6.2). After centrifugation of the mixture at 70× *g* for 15 min (min) at room temperature (RT), supernatant was collected and centrifuged at 600× *g* for 5 min at RT. The resulting platelet pellet was resuspended in buffer B (pH 7.4) for platelet aggregation and ex vivo phosphorylation experiments. For Western Blot experiments, (co-)immunoprecipitation and in vitro phosphorylation, platelet pellet was homogenized directly after isolation with 2% Lubrol-buffer (2% nonaethylene glycol monododecyl ether, 150 mM NaCl, 20 mM Tris in ddH_2_O, pH 8.0), containing protease inhibitors (1 mM benzamidine, 0.5 µg/mL leupeptin, 300 µM PMSF) and 1× PhosSTOP (Roche, Mannheim, Germany). The homogenate was centrifuged at 18,000× *g* for 15 min at 4 °C to remove cell debris and supernatant was collected and stored at −80 °C until further experiments were conducted. Protein concentration was detected using a Lowry kit (Bio-Rad Laboratories, Inc., Munich, Germany).

### 4.4. In Vitro Phosphorylation of Platelets

For detecting IRAG2 phosphorylation in platelets, 500 µg of lysate was incubated in 200 µL phosphorylation buffer (50 mM MES, 10 mM ATP, pH 7.2, protease and phosphatase inhibitors as described in Section 4.3) and stimulated with 100 µM 8-Br-cGMP for 20 min at 37 °C or ddH_2_O as a control. After phosphorylation, lysates were kept on ice and IRAG2 was immunoprecipitated by IRAG2 antibody as described in Section 4.6 and analyzed by SDS-Page and Western Blot. Phosphorylation of immunoprecipitated IRAG2 was detected via phospho-(Ser/Thr) PKA substrate antibody.

### 4.5. Ex Vivo Phosphorylation of Intact Platelets

An amount of 1.75 × 10^8^ platelets from IRAG2-WT or IRAG2-KO were preincubated in 500 µL of buffer B (pH 7.4) for 1 h at 37 °C, followed by incubation with 100 µM 8-pCPT-cGMP for 20 min at 37 °C or ddH_2_O as a control. After stimulation, platelets were centrifuged at 2000× *g* for 5 min at 4 °C and platelet pellet was homogenized with 150 µL of 2% Lubrol buffer (Section 4.3). The homogenate was centrifuged at 18,000× *g* for 15 min at 4 °C and supernatant was collected. An amount of 350 µL of co-immunoprecipitation buffer (Section 4.6) was added and immunoprecipitation of IRAG2 was carried out as described in Section 4.6 to detect IRAG2-specific phosphorylation.

### 4.6. (Co)-Immunoprecipitation

(Co)-immunoprecipitation was carried out as described before [17]. Briefly, 1000 µg of platelet lysates or 500 µg of phosphorylated platelet lysates were incubated in 500 µL of co-immunoprecipitation buffer (50 mM Tris HCl, 15 mM EGTA, 100 mM NaCl, 0.1% Triton X-100 in ddH_2_O, pH 7.5) containing protease inhibitors, 1× PhosSTOP (Roche, Mannheim, Germany), 1 mM DTT and 1 µg of anti-mouse LRMP (C-terminal) antibody = anti-IRAG2 antibody for 90 min on ice. Following incubation, mixture was centrifuged at 18,000× *g* for 10 min at 4 °C and supernatant was transferred to 15 µL of washed and blocked (3% BSA in co-immunoprecipitation-buffer, 30 min, 4 °C, Protein-A-Sepharose-Beads (Sigma-Aldrich^®^, Taufkirchen, Germany) and incubated overnight at 4 °C. Beads were eluted with 2× Laemmli buffer and supernatant was analyzed by SDS-Page and Western Blot (Section 4.7).

### 4.7. Western Blot Analysis

Expression of several proteins was analyzed via Western Blot. Therefore, 35 to 70 µg of protein was used for SDS-Page and blotted on a PVDF membrane (Immobilon-P, Merck KGaA, Darmstadt, Germany) after electrophoresis. Membranes were blocked with 5% non-fatty milk (VWR, Darmstadt, Germany), containing 0.05% Tween^®^ 20 (Sigma-Aldrich^®^, Taufkirchen, Germany) for 2 h at RT, followed by incubating the membrane with primary antibodies (anti-mouse LRMP (C-terminal) = anti-IRAG2: 1:1000; anti-IP_3_R1: 1:1000; anti-IP_3_R2: 1:200; anti-IP_3_R3: 1:100; anti-β-Galactosidase: 1:1000; anti-IRAG1: 1:200; anti-PKGIβ: 1:200; anti-phospho-(Ser/Thr) PKA substrate: 1:1000) at 4 °C overnight. After incubating the membranes in HRP-conjugated secondary antibodies (goat-anti-mouse: 1:10,000; goat-anti-rabbit: 1:10,000; donkey-anti-goat: 1:10,000; goat-anti-chicken: 1:5000) for 2 h at RT, detection was carried out using Clarity^TM^ Western ECL Substrate (Bio-Rad Laboratories, Inc., Munich, Germany) and ChemiDoc^TM^ MP Imaging System (Bio-Rad Laboratories, Inc., Munich, Germany). For statistical analysis, the signal intensity of each band was quantified using Image Lab^TM^ Software (Bio-Rad Laboratories, Inc., Munich, Germany) and was normalized to the total protein of the appropriate lane. Therefore, 1.5% trichloroethanol-containing hand-casting SDS-gels were used. Analyzed number of lysates from different animals are indicated by n-numbers in the description of the figure. Quantified samples were applied to one up to three gels (depending on the analyzed n-number) and were evaluated after the same gel-run at the same timepoints.

### 4.8. Platelet Aggregation

Platelets from IRAG2-WT and IRAG2-KO mice (each 1.0 × 10^8^ platelets/mL) were preincubated in buffer B (20 mM HEPES, 138 mM NaCl, 2.9 mM KCl, 1 mM MgCl_2_, 0.36 mM NaH_2_PO_4_, pH 7.4) for 5 min at 37 °C without stirring and then incubated with or without 8-pCPT-cGMP (150 µM or 200 µM) for 10 min at 37 °C, SNP (2.5 µM) for 2 min at 37 °C or buffer B in equal amounts as a control. Following that, aggregation was initiated by collagen (5 µg/mL) or thrombin (0.024 U/mL, 0.05 U/mL or 0.1 U/mL). Aggregation was measured at 37 °C with stirring (1200 rpm) by an optical aggregometer (Chronolog, Havertown, PA, USA) using the “Aggro/Link Software 5.1”. Results were calculated as maximal slope.

### 4.9. Statistical Analysis

All data are shown as mean ± SEM. For testing the samples for normality, the Shapiro–Wilk test was used. Normally distributed parameters were analyzed using an unpaired Student’s t-test to calculate significant differences between two means. Non-parametric data were analyzed using the Wilcoxon–Mann–Whitney test. Statistical analysis was performed with “GraphPad Prism version 5.01”. Significant differences in the graphs are shown by asterisks (*) (*p* < 0.05), (**) (*p* < 0.01) and (***) (*p* < 0.001).

## Data Availability

The datasets for this manuscript are not publicly available because the raw data supporting the conclusions of this manuscript will be made available by the authors, without undue reservation, to any qualified researcher. Requests to access the datasets should be directed to the corresponding author: jens.schlossmann@chemie.uni-regensburg.de.

## Supplementary Materials

The following supporting information can be downloaded at: https://www.mdpi.com/article/10.3390/ijms23126695/s1.
