# Supplementary material for: Function of IRAG2 Is Modulated by NO/cGMP in Murine Platelets
Source: Int J Mol Sci. 2022 Jun 15;23(12):6695. doi: 10.3390/ijms23126695 (PMC9223716; doi:10.3390/ijms23126695)
Supplement: Supplementary file 1 [file ijms-23-06695-s001.zip › ijms-1755660-Supplement.pdf]

Supplementary Material

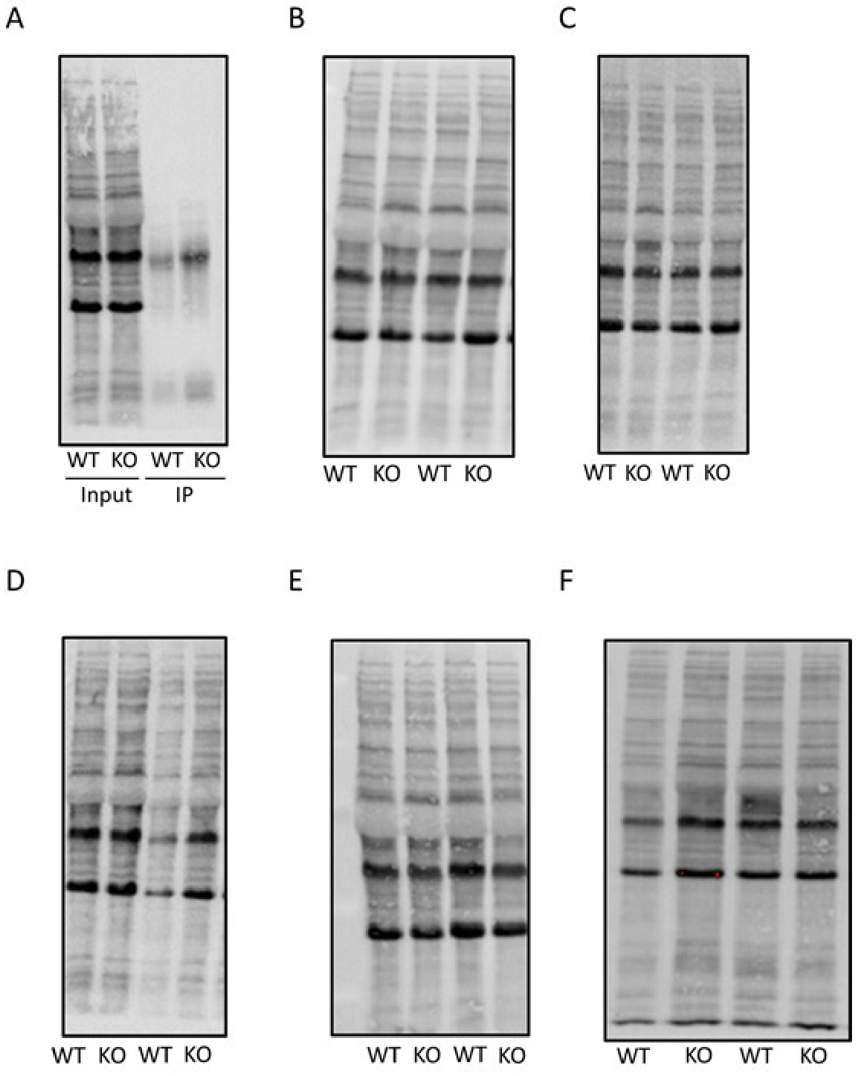

Figure S1. Total protein images of platelets from co-immunoprecipitation **(A)** and analysis of IP_3R1 **(B)**, IP_3R2 **(C)**, IP_3R3 **(D)**, IRAG1 **(E)** and PKGIβ **(F)** expression.

Total protein images from Western Blots for co-immunoprecipitation (Figure 2A) and analysis of expression of IP_3R1 (Figure 2B), IP_3R2 (Figure 2C), IP_3R3 (Figure 2D), IRAG1 (Figure 2E) and PKGIβ (Figure 2F) in platelets from IRAG2-WT (WT) and IRAG2-KO (KO) mice. Input per lane: 70 μg of protein for analysis of IRAG1, IP_3R1, IP_3R2 and IP_3R3 expression and 35 μg of protein for PKGIβ expression. For co-immunoprecipitation 1000 μg of murine platelet lysate was used.

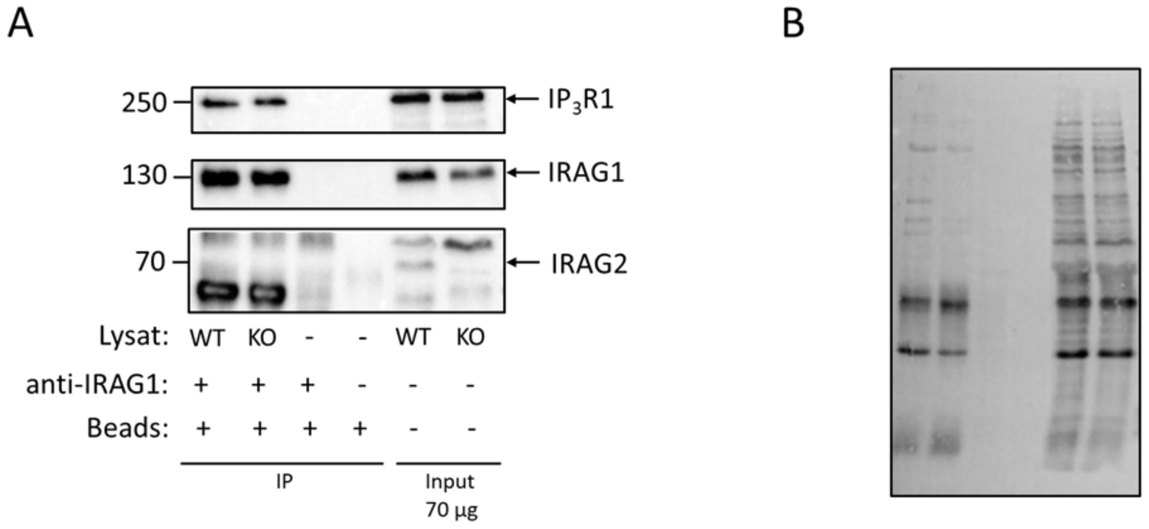

Figure S2. Immunoprecipitation of IRAG1 with IRAG1 specific antibody in platelets from IRAG2-WT (WT) and IRAG2-KO (KO) mice and Western Blot analysis of co-immunoprecipitated interaction partners. **(A)** Interaction of IRAG1 with IP3R1 and PKGIβ in murine platelets was detected in IRAG2-WT (WT) and IRAG2-KO (KO) platelets. No direct interaction of IRAG1 and IRAG2 was detected neither in platelets from IRAG2-WT (WT) nor in IRAG2-KO (KO) platelets. (**B**) Total protein image from Western Blot for co-immunoprecipitation (**A**) is shown. As input probes for IRAG2-WT (WT) and IRAG2-KO (KO) platelets 70 µg of protein and for immunoprecipitation 1000 µg of platelet lysate was used.

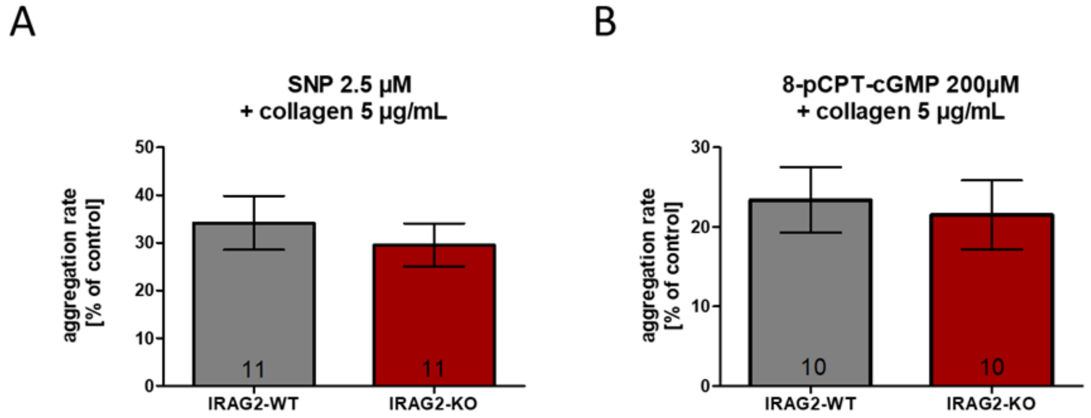

Figure S3: NO/cGMP-dependent inhibition of platelet aggregation in platelets from IRAG2-WT and IRAG2-KO mice using SNP 2.5 µM (**A**) or 8-pCPT-cGMP 200 µM (**B**) as an inhibitor and collagen 5 µg/mL to evoke platelet aggregation. Aggregation rate was calculated as maximal slope of the aggregation curve and aggregation rate under treatment with inhibitor is calculated as % of control (control: aggregation rate without inhibitor). Only a weak tendency for a reduced aggregation rate [% of control] was seen in IRAG2-KO platelets compared to IRAG2-WT platelets, which is not significant. For each aggregation 1.0×10^8 platelets/ml were used. The number of experiments is shown in the graphs and mean ± SEM is shown by bars.
